# Effect of dolutegravir-based versus efavirenz-based antiretroviral therapy on excessive weight gain in adult treatment-naïve HIV patients at Matsanjeni health center, Eswatini: a retrospective cohort study

**DOI:** 10.1186/s12981-023-00591-3

**Published:** 2024-01-07

**Authors:** Didier M. Mukuna, Tom Decroo, Clara M. Nyapokoto

**Affiliations:** 1 Ministry of Health, Matsanjeni Health Centre, ART Department, Matsanjeni, Eswatini; 2 Unit of HIV and TB, Department of Clinical Sciences, Institute of Tropical Medicine, Antwerp, Belgium; 3 Eswatini National AIDS Program, Mbabane, Eswatini

**Keywords:** Excessive weight gain, Dolutegravir, Efavirenz, Matsanjeni health centre

## Abstract

**Background:**

There is limited data on dolutegravir (DTG)-associated weight gain from settings with a dual burden of HIV and overnutrition.

**Methods:**

In Eswatini (at Matsanjeni), among 156 and 160 adult patients on DTG-based and EFV-based antiretroviral therapy (ART), respectively, we studied excessive weight gain (BMI at 24 months ART greater than baseline and ≥25 kg/m^2^).

**Results:**

The median BMI increase in DTG-based patients was 1.09 (IQR:-0.28,3.28) kg/m^2^ compared to 0.20 (IQR:-0.85,2.18) kg/m^2^ in EFV-based patients (*p* value = 0.001). DTG-based ART predicted excessive weight gain (aOR 2.61;95% CI:1.39–4.93).

**Conclusion:**

Practitioners should consider DTG-based regimens as one of the risk factors for overweight/obesity.

## Introduction

WHO recommends dolutegravir (DTG)-based antiretroviral therapy (ART) because of its efficacy, tolerability and high genetic resistance barrier [1]. However, despite its efficacy, there is evidence of excessive body weight increase associated with DTG-based regimens, especially when DTG is combined with tenofovir alafenamide-based than with tenofovir disoproxil fumarate (TDF)-containing backbones or other nucleoside reverse transcriptase inhibitors [2]. Moreover, several concerns about this excessive weight gain and its associated cardiometabolic complications have emerged, whereas people living with HIV (PLHIV) face rising morbidity and mortality from noncommunicable diseases (NCDs) [3].

Eswatini has the world’s highest HIV prevalence, with 25.9% among adults aged 15 to 49 years [4]. Furthermore, in Sub-Saharan Africa, Eswatini has one of the highest prevalences of overweight (27.7%) and obesity (23.0%) in women aged 15–49 years [5]. In Eswatini, as in other low- and middle-income countries (LMICs), while DTG use is increasing, data on DTG-associated weight gain are still limited [6]. At Matsanjeni Health Center (MHC), we therefore compared the effect of DTG-based versus EFV-based regimens on excessive weight gain 24 months after starting ART in adult treatment-naïve HIV patients.

## Methods

### Design

This retrospective cohort study used data routinely collected between 1 January 2016 and 31 December 2020.

### Setting

Eswatini has an estimated population of 1 146 903. Eswatini is one of the few countries that has met the triple 95% UNAIDS targets [7]. The MHC is a secondary health facility located in a rural area of southern Eswatini that is characterised by poverty, food insecurity and low level of education. This environment encourages vulnerable households to engage in risky behaviours that could expose them to HIV.

### Study population

We recruited all adult treatment-naïve patients, ≥18 years old, who started ART between 1 January 2016 and 31 December 2020, and remained in care for at least 24 months. We excluded pregnant women, patients with type 2 diabetes mellitus or tuberculosis, those who interrupted ART for 30 days or more, and patients whose regimen was switched.

### Variables: definitions and data collection

We calculated the BMI (kg/m^2^) from weight in kilograms (kg) and height in meters (m). We trained staff on how to measure weight and height and calculate BMI. We categorised BMI as underweight (< 18.5 kg/m^2^), normal (18.5–24.9 kg/m^2^), overweight (25-29.9 kg/m^2^) and obese (≥30 kg/m^2^). We categorised obesity as class 1 (30-34.9 kg/m^2^), class 2 (35-39.9 kg/m^2^) and class 3 (≥40 kg/m^2^). We defined excessive weight gain as any BMI greater than the baseline BMI category and ≥25 kg/m^2^ 24 months after ART initiation.

Study nurses encoded data into a case report form (CRF). The data clerk and the principal investigator performed a double entry of data from CRF to the study database (Microsoft Excel), after which verification and cleaning was performed.

### Statistics

We performed the Wilcoxon rank sum test to compare the median BMI between groups (DTG-based versus EFV-based ART). Multivariable logistic regression was used to determine the effect of ART on excessive weight gain 24 months after ART initiation, adjusted for confounding factors.

### Ethics

The institutional review board of the Institute of Tropical Medicine in Antwerp and the Eswatini Health and Human Research Review Board (EHHRRB) approved the study (references 1576/22 and EHHRRB032/2022, respectively). We obtained a waiver of informed consent from the EHHRRB.

## Results

**Fig. 1:**
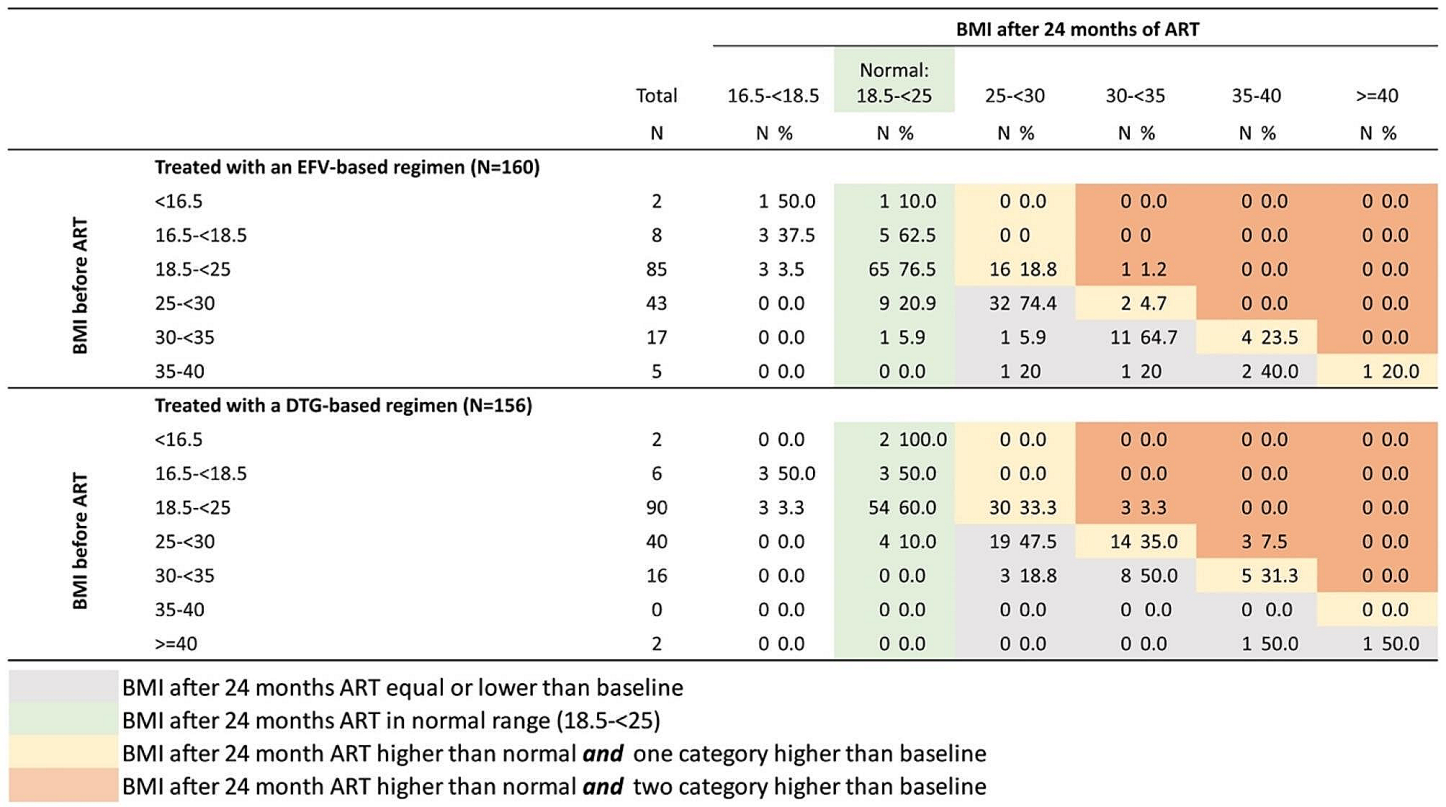
BMI at baseline versus BMI at 24 months, stratified by type of ART regimen

After applying inclusion and exclusion criteria, 317 patients were eligible. One was excluded because BMI data were incomplete. Of the remaining 316, 160 patients were on an EFV-based regimen and 156 patients on a DTG-based regimen. Figure 1 shows the evolution of BMI category after 24 months of ART, stratified by regimen and by baseline BMI category.

Overall, the median change in BMI in those treated with the DTG-based regimen was 1.09 (IQR: -0,28–3.28) kg/m^2^ compared to 0.20 (IQR: -0.85–2.18) kg/m^2^ among those receiving an EFV-based regimen, a statistically significant difference (*p* value = 0.001). An important proportion (36.7%; 33/90) of patients on the DTG-based regimen progressed from normal BMI at baseline to overweight, including 3 progressing to obesity class 1. Among 85 with normal BMI at baseline and on an EFV-based regimen, 20.0% (17/85) evolved to overweight *(see* Fig. 1*)*.

Overall, and 24 months after starting ART, 25% (79/316) of patients experienced an excessive increase in BMI. After controlling for confounding factors, patients taking the DTG-based regimen were more likely to experience an excessive BMI increase than those taking the EFV-based regimen (aOR 2.61;95%CI:1.39–4.93; *see* Table 1).

**Table 1:** Effect of ART regimen on excessive BMI increase, $ adjusted for confounding factors

	No Excessive BMI increase		Excessive BMI increase		aOR	[95%CI]
	N	%	N	%		
Total	237	75.0	79	25.0		
Gender					NS	
Female	153	78.5	42	21.5		
Male	84	69.4	37	30.6		
Age (median, IQR)	37	(31–45)	38	(30–34)	NS	
Economic status					NS	
Middle	12	60.0	8	40.0		
Low	225	76.0	71	24.0		
Educational level					NS	
Primary	138	79.8	35	20.2		
Secondary	93	69.9	40	30.1		
Tertiary	6	60.0	4	40.0		
CD4 at baseline (median, IQR)	343	(215–462)	297	(142–476)	NS	
WHO STAGE					NS	
1	139	74.7	47	25.3		
2	75	79.8	19	20.2		
3	21	65.6	11	34.4		
4	2	50.0	2	50.0		
Regimen						
EFV-based	136	85.0	24	15.0	1	
DTG-based	101	64.7	55	35.3	2.61**	[1.39,4.93]

EFV: efavirenz; DTG: dolutergravir; BMI:body mass index; aOR: adjusted odds ratio;

IQR: interquartile range; NS: not significant; * *p* < 0.05, ** *p* < 0.01, *** *p* < 0.001

^*$*^ BMI excessive increase: increase at 24 months ART of at least one category compared to baseline, and with BMI > = 25 at 24 months ART

## Discussion

WHO recommends a DTG-based regimen as the preferred first- or second-line regimen for ART. DTG-based regimens are highly effective. In this retrospective cohort study, we showed that excessive BMI increase was observed in 25% of participants 24 months after treatment initiation. Patients on the DTG-based regimen had a significantly higher BMI increase than those on the EFV-based regimen. Our findings are coherent with previous research, showing significant weight gain when ART-naïve patients began DTG-based therapy in the United States [8, 9]. It has also been observed in patients who switched from a non-INSTI-based to an INSTI-based regimen in some LMICs and upper-middle-income countries such as Thailand [10, 11].

While in many previous studies weight gain was quantified as kilograms gained over time [9, 12], Calza et al. and Esber et al. investigated weight gain as a number of BMI units over time. Calza et al. showed a mean increase in BMI of 0.84 kg/m^2^, observed in DTG-based treated patients at month 12 post ART initiation (*p* value > 0.05) [13]. Differences between his findings and ours may be explained by differences in the study population characteristics and the length of the follow-up period. Indeed, Calza et al. investigated weight gain using a holistic approach, including BMI, after 12 months of treatment in ART-naïve HIV patients starting an INSTI-based or darunavir/ritonavir-based regimen, among whom the vast majority were Caucasian, while black race had been identified as one of the risk factors for weight gain in many other studies [8, 10]. However, our results, showing a higher increase at 24 months for DTG-based than for EFV-based regimens, complements those of Esber et al.who showed an annual mean change in BMI at one year of 1.25 kg/m^2^ [6].

In Eswatini at present, more than 80% of PLHIV on ART receive DTG-based therapy. Considering that 25% of patients in our study had an excessive BMI increase, and the dual burden of HIV and overnutrition in Eswatini, our findings underscore the importance of educating patients about the risk of overweight/obesity and nonpharmacological interventions such as diets and physical exercise when initiating DTG-based therapy. When deciding which ART regimen is most appropriate for a patient, clinicians should know that ART regimens can have an effect beyond mere viral load suppression and may result, or not, in body weight maintenance [10]; they should therefore consider the patient’s baseline BMI and have a clinical and laboratory monitoring plan in place to prevent obesity and its cardiometabolic complications.

Our study was the first of its kind at MHC. It was carried out using routinely collected data, and reflects the completeness and accuracy with which the ART clinic from MHC has collected data. Therefore, it encourages other healthcare organisations to own and generate quality data for clinical decision-making.

Our study had some limitations. This is a retrospective observational study of routinely collected data. Given this design, we could only show an assocation but not assess a possible causal relationship between the use of DTG versus EFV and excessive weight gain. We collected the main variables of interest (height, weight, and BMI) for this study from patients records. However, data was lacking for some relevant factors, such as history of hypertension, lifestyle, waist circumference, lipid profile, and blood glucose. Most patients had a low socio-economic status and educational level. This is because all the participants came from MHC, a health centre in a remote area where most people are known to be poor and illiterate. Therefore, our results may not be generalizable to PLHIV from other settings.However, the findings are coherent with those from other studies conducted in black populations [8, 10].

In conclusion, in our cohort, 24 months after starting therapy, excessive BMI increase was significantly higher among patients on a DTG-based compared with an EFV-based regimen. DTG-based therapy will remain the preferred ART regimen in Eswatini, due to its effectiveness and circulating resistance to NNRTIs. However, DTG-based ART should be considered as a risk factor for overweight/obesity in PLHIV. To prevent obesity-associated NCDs, using an upstream approach, clinicians should consider the patient’s baseline BMI and have a clinical and laboratory monitoring plan in place. Moreover, the Eswatini national AIDS program should develop guidelines for clinical and laboratory monitoring of weight and management of obesity, including rules for ART switching, to reduce the risk of cardiometabolic complications associated with obesity.

## Data Availability

The data that support the findings of this study are available from the corresponding author but restrictions apply to the availability of these data, which were used under license for the current study, and so are not publicly available. Data are however available from the corresponding author upon reasonable request and with permission of the Ministry of Health.

## Abbreviations

AIDS: Acquired immunodeficiency syndrome
aOR: Adjusted odds ratio
ART: Antiretroviral therapy
BMI: Body mass index
CI: Confidence interval
CRF: Case report form
DTG: Dolutegravir
EFV: Efavirenz
EHHRRB: Eswatini Health and Human Research Review Board
HIV: Human immunodeficiency virus
INSTI: Integrase strand transfer inhibitor
IQR: Interquartile range
LMICs: Low- and middle-income countries
MHC: Matsanjeni Health Center
NCDs: Noncommunicable diseases
NNRTIs: Non-nucleoside reverse transcriptase inhibitors
NS: Not significant
PLHIV: People living with HIV
TDF: Tenofovir disoproxil fumarate
UNAIDS: Joint United Nations Programme on HIV/AIDS
WHO: World health organization
